# Mid-Infrared Laser Generation of Zn_1−x_Mn_x_Se and Zn_1−x_Mg_x_Se (x ≈ 0.3) Single Crystals Co-Doped by Cr^2+^ and Fe^2+^ Ions—Comparison of Different Excitation Wavelengths

**DOI:** 10.3390/ma15155277

**Published:** 2022-07-30

**Authors:** Adam Říha, Helena Jelínková, Maxim E. Doroshenko, Michal Jelínek, Jan Šulc, Michal Němec, David Vyhlídal, Nazar O. Kovalenko

**Affiliations:** 1 Faculty of Nuclear Sciences and Physical Engineering (FNSPE), Czech Technical University in Prague, Břehová 7, 115 19 Prague, Czech Republic; helena.jelinkova@fjfi.cvut.cz (H.J.); michal.jelinek@fjfi.cvut.cz (M.J.); jan.sulc@fjfi.cvut.cz (J.Š.); michal.nemec@fjfi.cvut.cz (M.N.); david.vyhlidal@fjfi.cvut.cz (D.V.); 2 Prokhorov General Physics Institute, Vavilov Str. 38, 119991 Moscow, Russia; dorosh@lst.gpi.ru; 3 Institute for Single Crystals, NAS of Ukraine, Nauky Ave. 60, 61001 Kharkiv, Ukraine; nazar@isc.kharkov.ua

**Keywords:** mid-infrared laser, solid-state laser, ions energy transfer, Cr,Fe:ZnMnSe, Cr,Fe:ZnMgSe

## Abstract

Two different mid-infrared (mid-IR) solid-state crystalline laser active media of Cr${}^{2+}$, Fe${}^{2+}$:Zn${}_{1-x}$Mn${}_{x}$Se and Cr${}^{2+}$, Fe${}^{2+}$:Zn${}_{1-x}$Mg${}_{x}$Se with similar amounts of manganese or magnesium ions of x ≈ 0.3 were investigated at cryogenic temperatures for three different excitation wavelengths: Q-switched Er:YLF laser at the wavelength of 1.73 μm, Q-switched Er:YAG laser at 2.94 μm, and the gain-switched Fe:ZnSe laser operated at a liquid nitrogen temperature of 78 K at ∼4.05 μm. The temperature dependence of spectral and laser characteristics was measured. Depending on the excitation wavelength and the selected output coupler, both laser systems were able to generate radiation by Cr${}^{2+}$ or by Fe${}^{2+}$ ions under direct excitation or indirectly by the Cr${}^{2+}$→ Fe${}^{2+}$ energy transfer mechanism. Laser generation of Fe${}^{2+}$ ions in Cr${}^{2+}$, Fe${}^{2+}$:Zn${}_{1-x}$Mn${}_{x}$Se and Cr${}^{2+}$, Fe${}^{2+}$:Zn${}_{1-x}$Mg${}_{x}$Se (x ≈ 0.3) crystals at the wavelengths of ∼4.4 and ∼4.8 μm at a temperature of 78 K was achieved, respectively. The excitation of Fe${}^{2+}$ ions in both samples by direct 2.94 μm as well as ∼4.05 μm radiation or indirectly via the Cr${}^{2+}$→ Fe${}^{2+}$ ions’ energy transfer-based mechanism by 1.73 μm radiation was demonstrated. Based on the obtained results, the possibility of developing novel coherent laser systems in mid-IR regions (∼2.3–2.5 and ∼4.4–4.9 μm) based on A${}^{\mathrm{II}}$B${}^{\mathrm{VI}}$ matrices was presented.

## 1. Introduction

There is a continuous demand for affordable and reliable sources of middle infrared (mid-IR) laser radiation usable for all kinds of applications, including spectroscopy, medical treatment, biomedical non-invasive diagnostics, remote sensing of atmospheric constituents (trace gases), chemical and biological analysis, metrology, optical radars, optical communication in free space, optical switching, laser radar in eye-safe range, military applications (target designation or obstacle avoidance), and others [1,2,3,4]. The dominant spectroscopic measurements are based on strong molecular rotational and vibrational transitions in a molecular fingerprint region from 2 to 25 μm [1]. Moreover, these lasers can be utilized for the pumping of nonlinear laser materials (i.e., OPOs) [3].

A promising way, among others, is to utilize the A${}^{\mathrm{II}}$B${}^{\mathrm{VI}}$ chalcogenides doped with divalent laser active transition metal ions (TM${}^{2+}$). The groups of DeLoach, Adams and Page et al. [5,6,7] introduced a brand new class of mid-IR Cr${}^{2+}$ and Fe${}^{2+}$ ions doped with II–VI laser active media during the second half of the 1990s. Since then, lasers based on these ions doped into ZnS [8,9], ZnSe [7,10,11,12], CdSe [13], and recently also into Cd${}_{1-x}$Mn${}_{x}$Te [14,15,16], Zn${}_{1-x}$Mn${}_{x}$Se [17,18,19], Zn${}_{1-x}$Mn${}_{x}$Te [20,21], Zn${}_{1-x}$Mg${}_{x}$Se [22,23], and other crystals generated in continuous wave (CW) [24,25], free-running (pumped by laser diodes [26,27,28] or fiber lasers [26,27,28]), Q-switched, and mode-locking [12,29,30] regimes of operation have been reported. Moreover, these lasers are tunable over a wide spectral range of ∼2–3.5 μm in the case of Cr${}^{2+}$ ions [31] or over ∼3.7–5.1 μm for the Fe${}^{2+}$ ions doped with ZnSe [32].

In several previous studies [28,32,33,34,35,36,37,38], the quite efficient energy transfer of Cr${}^{2+}$ → Fe${}^{2+}$ in Cr,Fe:Zn${}_{1-x}$Mn${}_{x}$Se materials was confirmed. The first demonstration of the Cr${}^{2+}$ → Fe${}^{2+}$ ions’ energy transfer in Cr,Fe:Zn${}_{1-x}$Mn${}_{x}$Se (x ≈ 0.3) obtained in temperatures up to 150 K was presented in [34]. This article offers a comparison of these results with that recently obtained in a different host material with a similar amount of magnesium instead of manganese in the host. Both crystals are characterized by a similar amount of Mn and Mg in the solid solution. However, the Mg crystal at this Mg concentration (x ≈ 0.3) is characterized by the different crystal structure (wurtzite/hexagonal) instead of Mn crystal, which crystallizes in the zinc blend/cubic structure at this Mn concentration. Therefore, this paper compares principally different crystal structures. Moreover, some novel results of the Cr,Fe:Zn${}_{1-x}$Mn${}_{x}$Se (x ≈ 0.3), for example, excitation at wavelengths of 2.94 and ∼4.05 μm, are included.

In this paper, the possibility of the energy transfer excitation mechanism for Cr,Fe:Zn${}_{1-x}$Mn${}_{x}$Se (x ≈ 0.3) and Cr,Fe:Zn${}_{1-x}$Mg${}_{x}$Se (x ≈ 0.3) crystal samples is reported. Laser operations of both laser systems under three different excitation wavelengths were realized and compared. This paper also contains new results of the Cr,Fe:Zn${}_{1-x}$Mg${}_{x}$Se (x ≈ 0.3) excitation at ∼4.05 μm radiation compared to [38], and the text has been extended by a section analyzing and comparing the fluorescence decay time and energy transfer efficiency with its calculation methods. The temperature dependences of spectroscopic and laser characteristics of both active laser media for different coherent radiation excitation at 1.73, 2.94, and ∼4.05 μm are presented and compared.

## 2. Materials and Methods

**Figure 1:**
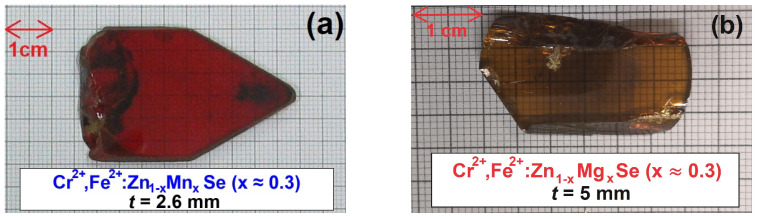
Photos of polished single crystal samples with a 1 cm scale. (**a**) Cr,Fe:Zn${}_{1-x}$Mn${}_{x}$Se (x ≈ 0.3) crystal. (**b**) Cr,Fe:Zn${}_{1-x}$Mg${}_{x}$Se (x ≈ 0.3) crystal.

The Cr,Fe:Zn${}_{1-x}$Mn${}_{x}$Se and Cr,Fe:Zn${}_{1-x}$Mg${}_{x}$Se (x ≈ 0.3) crystalline active media were synthesized by the high-pressure Bridgman method with active transition metal ions of Cr${}^{2+}$ and Fe${}^{2+}$ doping during the synthesis process. The concentrations of Cr${}^{2+}$ and Fe${}^{2+}$ active ions in the Mn sample were C(Cr${}^{2+}$) ≈ 3 × 10${}^{18}$ cm${}^{-3}$, and C(Fe${}^{2+}$) ≈ 5 × 10${}^{18}$ cm${}^{-3}$. The Mg crystal was similarly grown from the melt that consisted of 4 × 10${}^{-3}$ weight % of Cr${}^{2+}$ ions in the whole melt and of 2 × 10${}^{-2}$ weight % of Fe${}^{2+}$ ions. It means that the concentrations were C(Cr${}^{2+}$) ≈ 0.6 × 10${}^{18}$ cm${}^{-3}$, and C(Fe${}^{2+}$) ≈ 3 × 10${}^{18}$ cm${}^{-3}$. The thicknesses of the polished samples were 2.6 and 5 mm, respectively. The crystal facets were optically polished without any antireflection coatings. Therefore, due to a relatively high refraction index of ∼2.5, the corresponding Fresnel reflection losses of ∼17.6% at both air–crystal interfaces were considered. It should be mentioned that the solid solutions of Zn${}_{1-x}$Mn${}_{x}$Se and Zn${}_{1-x}$Mg${}_{x}$Se for x ≈ 0.3 are characterized by different crystal structures: zinc blend (cubic) and wurtzite (hexagonal), respectively. The fluorescence lifetime measurements were compared with the Cr${}^{2+}$-only doped samples with similar amounts of Mn or Mg in the host material. In the following text, the crystals are referred to as the Mn sample and the Mg sample for simplification and the data obtained are represented in figures by blue and red colors, respectively. The photos of both polished single crystal samples are shown in Figure 1.

The transmission spectra of both crystals were measured by the FT-IR spectrometer (Thermo Scientific Nicolet iS5, spectral range: 7800–350 cm${}^{-1}$, i.e., ∼1.3–28.5 μm). The absorption coefficients were recalculated according to the thicknesses of the samples.

The fluorescence spectra were measured using the single grating monochromator (Oriel 77250, grating model no. 77301, primary wavelength region: 1.45–2.2 μm; spectral range using a corresponding grating: 1.45–8 μm). The Fe${}^{2+}$ ions fluorescence (at the 8th diffraction order) was detected by the liquid-nitrogen-cooled (LN${}_{2}$-cooled) mercury-cadmium-telluride (MCT) photodetector (Judson-Teledyne J15D12). The Cr${}^{2+}$ ions’ fluorescence (at the fourth diffraction order) was measured using the PbS fixed gain photodetector (Thorlabs PDA30G-EC, spectral range: 1–2.9 μm, rise time: 250 μs). The fluorescence decay curves of Cr${}^{2+}$ ions of both samples were measured by the InAsSb/InAsSbP photodetector (PD36-05, spectral region: 1.5–3.8 μm; rise/fall time: 100 ns/15 ns) under direct Q-switched pumping pulse at the wavelength of 1.73 μm. The Fe${}^{2+}$ ions fluorescence lifetime was measured simultaneously for the same excitation using the fast HgCdTe (MCT) photodetector (Vigo PVI-6, spectral region: 2.8–6 μm; time constant: ≤ 80 ns). The detectors were connected to the oscilloscope Tektronix DPO4104 (4 channels, bandwidth: 1 GHz, maximal sample rate: 5 GS/s).

The crystal samples were gradually placed inside the evacuated LN${}_{2}$-cooled cryostat (Janis VPF-100) with uncoated CaF${}_{2}$ windows. Optical cavity mirrors were placed as close as possible outside the windows. The energy of the generated laser radiation was measured by the sensitive energy probe (Coherent J10MB-LE). The beam profiles were measured by the pyroelectric camera (Pyrocam-III, Spiricon). Laser oscillations spectra were measured by the same monochromator (Oriel 77250) together with the PbS photodetector (Cr${}^{2+}$ ions oscillations) or by the LN${}_{2}$-cooled MCT photodetector (Fe${}^{2+}$ ions oscillations). The calibrated band- or longwave-pass filters (Spectrogon or Thorlabs) were used for blocking the pumping radiation at the input of the corresponding detectors.

The properties of three different pumping lasers used for the direct excitation of Cr${}^{2+}$ or Fe${}^{2+}$ ions, or indirect excitation of Fe${}^{2+}$ via Cr${}^{2+}$ ions, are described in Section 2.1 “Pumping Laser Systems”.

### 2.1. Pumping Laser Systems

For the active sample excitation, three short-pulsed operated laser systems with different output wavelengths were used: (a) the electro-optically Q-switched flash-lamp pumped Er:YLF laser, (b) the electro-optically Q-switched flash-lamp pumped Er:YAG laser (both operated at room temperature—RT), and (c) the LN${}_{2}$-cooled gain-switched Fe:ZnSe laser (pumped by Er:YAG laser) at 78 K. The comparison of the pumping lasers’ properties, such as central oscillation wavelength, pulse duration at full width at half maximum (FWHM), pulse energy, and repetition rate, are presented in Table 1.

## 3. Results

The results of spectroscopic and laser output properties for 1.73, 2.94, and ∼4.05 μm laser excitation wavelengths follow in Section 3.1 and Section 3.2, respectively.

### 3.1. Cr,Fe:Zn${}_{1-x}$Mn${}_{x}$Se and Cr,Fe:Zn${}_{1-x}$Mg${}_{x}$Se (x ≈ 0.3) Spectroscopic Properties

The spectroscopic properties of both investigated materials in the temperature range of 78–300 K were measured. The results obtained for absorption and fluorescence measurements can be found in Section 3.1.1 and Section 3.1.2, respectively. The fluorescence decay time and energy transfer efficiency are described in Section 3.1.3.

#### 3.1.1. Absorption Spectra

**Figure 2:**
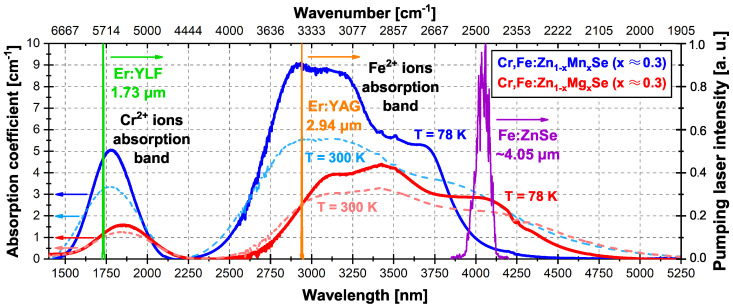
Absorption spectra of Cr,Fe:Zn${}_{1-x}$Mn${}_{x}$Se (x ≈ 0.3) (dark and light blue curves) and Cr,Fe:Zn${}_{1-x}$Mg${}_{x}$Se (x ≈ 0.3) samples (red and pink curves) at 78 K (full lines) and at 300 K (dashed lines). The green, orange, and purple curves show the pumping laser’s oscillation spectra at wavelengths of 1.73, 2.94, and ∼4.05 μm, respectively.

The absorption spectra of both crystal samples at 78 and 300 K, together with the used pumping laser’s oscillation spectra, are shown in Figure 2.

The relative ratio between Cr${}^{2+}$ and Fe${}^{2+}$ ions in the maxima of their absorption in the Mn and Mg samples were ∼0.55 and ∼0.35, respectively. If we compare this ratio at the excitation wavelengths used of 1.73 and 2.94 μm, the ratio was similar to ∼0.5 for both samples. The comparison of the Cr${}^{2+}$ and Fe${}^{2+}$ ions’ absorption bands width (FWHM) and maximum peak positions of investigated samples at 78 and 300 K are presented in Table 2. The measured results show that the solid solution containing magnesium has wider absorption bands of both active ions by a factor of ∼1.3, and the maxima of the absorption peaks are shifted toward longer wavelengths with respect to that in the Mn-based solid solution, which is comparable to the results published in [23]. The falling edge of the Fe${}^{2+}$ ions’ absorption band decreases more slowly in the case of the Mg sample in comparison to the relatively steep fall of this edge in the case of the Mn sample. The FWHM of the absorption bands of both crystals becomes wider with an increasing temperature. The absorption coefficient values of Mn as well as Mg samples for temperatures of 78 and 300 K at three different excitation wavelengths used, are summarized in Table 3.

#### 3.1.2. Fluorescence Spectra

**Figure 3:**
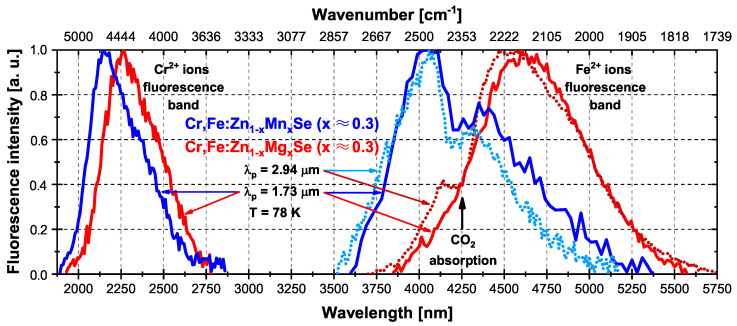
Fluorescence spectra of the Cr${}^{2+}$ and Fe${}^{2+}$ ions of Cr,Fe:Zn${}_{1-x}$Mn${}_{x}$Se (x ≈ 0.3) (blue) and Cr,Fe:Zn${}_{1-x}$Mg${}_{x}$Se (x ≈ 0.3) (red) crystals at a temperature of 78 K measured under direct excitation of Cr${}^{2+}$ ions and via the Cr${}^{2+}$→ Fe${}^{2+}$ ions’ energy transfer mechanism at the wavelength of 1.73 μm (full lines) and directly pumped Fe${}^{2+}$ ions at 2.94 μm (dotted lines).

Each sample has been inserted at an angle of 45° to the incident laser beam of the Er:YLF laser at 1.73 μm to obtain direct Cr${}^{2+}$ ions and/or indirect Fe${}^{2+}$ ions excitation via the Cr${}^{2+}$ → Fe${}^{2+}$ ion energy transfer or of Er:YAG laser at 2.94 μm to obtain directly excited Fe${}^{2+}$ ion fluorescence spectrum. In the case of the 1.73 μm radiation excitation, two simultaneous fluorescence signals at around ∼2.25 μm and in the range of 3.7–5.2 μm were measured. The normalized fluorescence spectra of both samples for the temperature of 78 K are presented in Figure 3. The atmospheric absorption drop in a measured signal, especially at ∼4.25 μm caused by CO${}_{2}$ gas molecules, was observed. As we can see from Figure 3, the Fe${}^{2+}$ ions’ fluorescence signal via the Cr${}^{2+}$ → Fe${}^{2+}$ ion energy transfer (dotted lines) corresponds well to the signal obtained under direct pumping by the wavelength of 2.94 μm.

**Figure 4:**
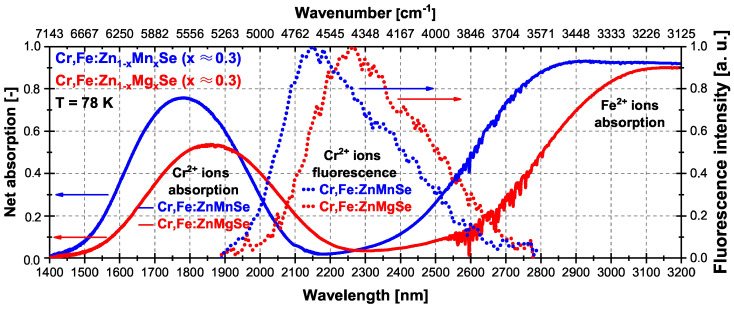
Net absorption spectra of Cr,Fe:Zn${}_{1-x}$Mn${}_{x}$Se (x ≈ 0.3) (full blue line) and Cr,Fe:Zn${}_{1-x}$Mg${}_{x}$Se (x ≈ 0.3) crystals (full red line) at 78 K together with the Cr${}^{2+}$ ions’ fluorescence spectra (dotted lines of same colors).

It is good to note here that the Cr${}^{2+}$ ions’ fluorescence spectra trailing edges were influenced by the presence of Fe${}^{2+}$ ions co-doped in these crystals and their absorption. Details of the Cr${}^{2+}$ ions’ fluorescence spectra together with the net absorption spectra of both samples at 78 K are presented in Figure 4.

**Figure 5:**
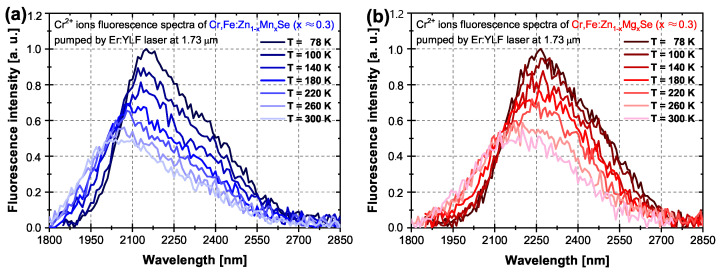
Temperature dependence of the Cr${}^{2+}$ fluorescence spectra excited directly at the wavelength of 1.73 μm. (**a**) Cr,Fe:Zn${}_{1-x}$Mn${}_{x}$Se x ≈ 0.3)—blue curves. (**b**) Cr,Fe:Zn${}_{1-x}$Mg${}_{x}$Se (x ≈ 0.3)—red curves.

The temperature dependence of the Cr${}^{2+}$ ions’ fluorescence of both samples is presented in Figure 5. As we can see, the spectrum intensity decreases with increasing temperature as the absorption coefficient decreases together with the shift of the fluorescence signal maximum towards shorter wavelengths. The maximal signals of the Cr${}^{2+}$ ions’ fluorescence in Mn and Mg samples were shifted from ∼2.15 μm at 78 K down to ∼2.05 μm at 300 K and from ∼2.26 μm at 78 K down to ∼2.17 μm at 300 K, respectively.

**Figure 6:**
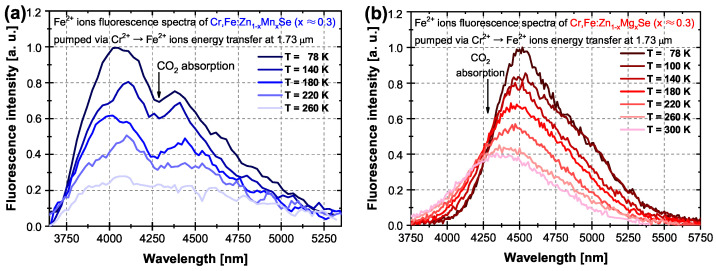
Temperature dependence of the Fe${}^{2+}$ fluorescence spectra excited via the Cr${}^{2+}$→ Fe${}^{2+}$ ion energy transfer at the wavelength of 1.73 μm. (**a**) Cr,Fe:Zn${}_{1-x}$Mn${}_{x}$Se (x ≈ 0.3)—blue curves. (**b**) Cr,Fe:Zn${}_{1-x}$Mg${}_{x}$Se (x ≈ 0.3)—red curves.

The Fe${}^{2+}$ ions’ fluorescence spectra temperature dependence of both samples is presented in Figure 6. As we can see, the spectrum intensity decreases with increasing temperature as the absorption coefficient decreases together with the shift of the fluorescence signal maximum towards shorter wavelengths. For the same content of Mn or Mg in the solid solution (x ≈ 0.3), the Fe${}^{2+}$ ions’ fluorescence in the Mg sample was shifted farther to mid-IR, and the gap between Cr${}^{2+}$ and Fe${}^{2+}$ ions’ fluorescence bands was wider. The maximal signals of the Fe${}^{2+}$ ions’ fluorescence in Mn and Mg samples were detected at ∼4.05 and ∼4.52 μm at 78 K, respectively.

#### 3.1.3. Fluorescence Decay Time and Energy Transfer Efficiency

**Figure 7:**
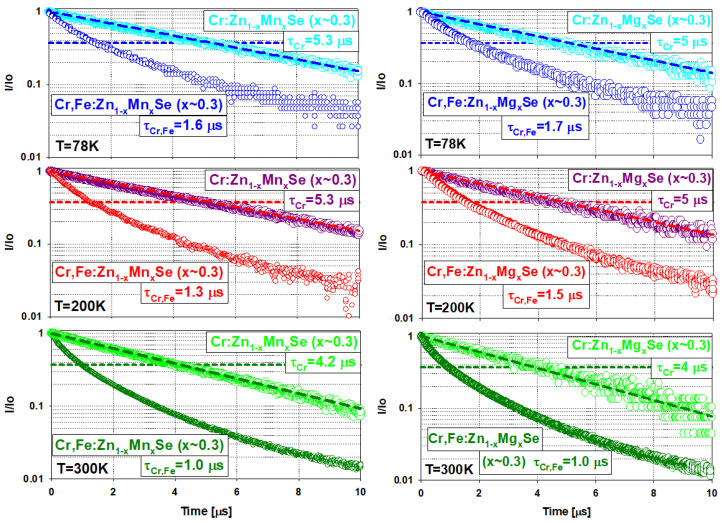
Temperature dependences of the Cr${}^{2+}$ ions’ fluorescence decay time in Cr,Fe:Zn${}_{1-x}$Mn${}_{x}$Se (x ≈ 0.3) and Cr,Fe:Zn${}_{1-x}$Mg${}_{x}$Se (x ≈ 0.3) crystal under ∼1.73 μm Q-switched laser excitation. $\tau_{\mathrm{Cr}}$—Cr${}^{2+}$ ions fluorescence lifetime of Cr${}^{2+}$-only doped crystal; $\tau_{\mathrm{Cr},\mathrm{Fe}}$ — Cr${}^{2+}$ ions’ decay time at the 1/e level of Cr${}^{2+}$,Fe${}^{2+}$ co-doped crystal.

In comparison with [37], some results obtained with novel excitation wavelengths are presented together with improved output laser parameters of the Mg sample. Moreover, in this section, we analyze and compare the fluorescence decay times and the energy transfer efficiency with its two calculation methods. In Figure 7, the examples of Cr${}^{2+}$ ions’ fluorescence decay curves in the Mn and Mg samples under 1.73 μm excitation are presented for different temperatures. As one can see in Figure 7, the decay curves are non-exponential for both crystals in the wide temperature range up to 300 K due to the Cr${}^{2+}$ → Fe${}^{2+}$ energy transfer process. For both crystals, the non-exponentiality trends increase with temperature. The single exponential fit at the decay curve’s tail also demonstrates the shortening of the Cr${}^{2+}$ ions’ lifetime in both Mn and Mg samples with a temperature increase similar to that shown in [22,39] for the Cr${}^{2+}$-only doped sample. The fluorescence lifetime of Cr${}^{2+}$ ions was calculated (at the decay curve’s tail) with values of ∼5.3 μs for the Mn samples and ∼5.0 μs for the Mg samples at 78 K, as shown in Figure 7 by the solid lines. The Cr${}^{2+}$ lifetime slowly decreases with a rising temperature to ∼4.2 and ∼4.0 μs at 300 K for the Mn and Mg samples, respectively. The dashed horizontal lines in Figure 7 show the 1/e level where the decay times of Cr${}^{2+}$ ions at different temperatures were determined.

Using the data in Figure 7, the Cr${}^{2+}$ → Fe${}^{2+}$ energy transfer efficiency $\eta_{T}$ was evaluated using the simple equation from [40]

(1) $$\eta_{T}=1-\frac{\tau_{\mathrm{Cr},\mathrm{Fe}}}{\tau_{\mathrm{Cr}}},$$

where $\tau_{\mathrm{Cr},\mathrm{Fe}}$ is the decay time determined at the 1/e level for the Cr${}^{2+}$ donor ion in the presence of the Fe${}^{2+}$ acceptor ion, and $\tau_{\mathrm{Cr}}$ is the lifetime of Cr${}^{2+}$ ions measured in the singly doped sample. The second calculation of energy transfer efficiency was based on integrals under Cr${}^{2+}$ ions’ decay curves

(2) $$\eta_{T}=1-\frac{\int_{0}^{\infty }I_{N}\left(t\right)\;dt}{\int_{0}^{\infty }I_{R}\left(t\right)dt},$$

where I${}_{N}$(*t*) is the intensity of Cr${}^{2+}$ decay in the singly doped crystal and I${}_{R}$(*t*) is the intensity of Cr${}^{2+}$ decay in the Cr${}^{2+}$,Fe${}^{2+}$ co-doped sample [34,40]. The results are summarized in Table 4.

As follows from Table 4, the values of energy transfer efficiency (calculated according to Equation (2)) for both crystals at 78 K are quite similar (∼55%) to those reported previously [34,38]. The energy transfer efficiency for both samples has a similar tendency to increase with temperature due to an increase in the overlap integral between the Cr${}^{2+}$ donor ions fluorescence and Fe${}^{2+}$ acceptor ions absorption though the evaluated efficiency in each case is higher for the Mn sample due to the higher doping concentration. It can also be noticed that a simple evaluation from Equation (1) using the 1/e level of the decay time gives, in both cases, the overestimated values, though the difference is decreased for room temperature. In the case of the fluorescence decay curve processing, more accurate measurement was performed at higher temperatures for both crystals, and thus, higher energy transfer efficiency was obtained in this case. However, the overall laser output efficiency is also affected by thermally activated non-radiative quenching, which sufficiently reduces the Fe${}^{2+}$ ions’ lifetime because Cr${}^{2+}$ ions quenched decay time $\tau_{\mathrm{Cr},\mathrm{Fe}}$ at room temperature (at 1/e level) for both crystals is about ∼1 μs. This makes the gain switch operation of Fe${}^{2+}$ ions (RT lifetime is about ∼100 ns) using Cr${}^{2+}$ → Fe${}^{2+}$ energy transfer quite a challenge. Therefore, the final laser efficiency can be much lower.

### 3.2. Cr,Fe:Zn${}_{1-x}$Mn${}_{x}$Se and Cr,Fe:Zn${}_{1-x}$Mg${}_{x}$Se (x ≈ 0.3) Lasers’ Output Properties

**Figure 8:**
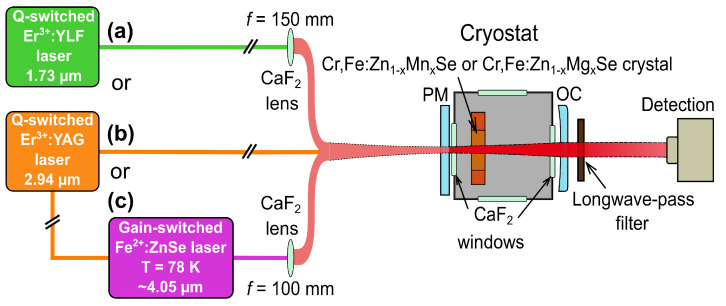
Cr,Fe:Zn${}_{1-x}$Mn${}_{x}$Se (x ≈ 0.3) and Cr,Fe:Zn${}_{1-x}$Mg${}_{x}$Se (x ≈ 0.3) laser systems’ experimental setup for three various pumping laser systems used: (a) Q-switched Er:YLF laser at the wavelength of 1.73 μm is directed to pumping, (b) Q-switched Er:YAG laser at 2.94 μm, and (c) gain-switched Fe:ZnSe laser at ∼4.05 μm operated for the temperature of 78 K pumped by Er:YAG laser is pumping Cr,Fe:Zn${}_{1-x}$Mn${}_{x}$Se (x ≈ 0.3) or Cr,Fe:Zn${}_{1-x}$Mg${}_{x}$Se (x ≈ 0.3) laser system.

The investigated laser active crystalline media were placed gradually in a copper holder inside the evacuated cryostat, which was then cooled down by liquid nitrogen to a temperature of 78 K. The laser output properties, such as laser output energy, pulse duration, oscillation spectra, and beam profile, were measured. The temperature was controlled by a Cryogenic Temperature Controller (model no. 325, Lake Shore Cryotronics). Three different excitation wavelengths of 1.73 (Section 3.2.1), 2.94, and ∼4.05 μm (Section 3.2.2) were applied subsequently. The experimental laser systems differed only in pumping systems and the set of cavity mirrors used. The simplified scheme is shown in Figure 8.

#### 3.2.1. Er:YLF Laser Excitation at 1.73 μm

For a better comparison, all investigated laser systems had similar cavity parameters—the laser cavity length of about ∼10 cm (placed close to the cryostat CaF${}_{2}$ windows) consisted of a pair of mirrors: a flat pumping mirror—PM and a curved output coupler—OC (*r* = −150 mm or *r* = −200 mm). To obtain the Cr${}^{2+}$ ions’ oscillation, the PM was highly reflective (HR) at 2.1–3.1 μm, and OC had a reflectivity of R${}_{\mathrm{OC}}$ ≥ 90% at 2.2–2.7 μm. In the case of the Cr${}^{2+}$ → Fe${}^{2+}$ ions pumping via the energy transfer process, the flat PM: HR at 3.9–5 μm (T ≈ 87% at 1.73 μm) and the OC with reflectivity R${}_{\mathrm{OC}}$ = 95% at 4.2–4.9 μm were used. In order to separate undesirable residual pump radiation, a Z-line (formed by a pair of mirrors (HR at 3.9–5 μm and T ≥ 85% at 1.7–2.7 μm)) as well as longwave-pass filters were used in front of the detectors.

**Figure 9:**
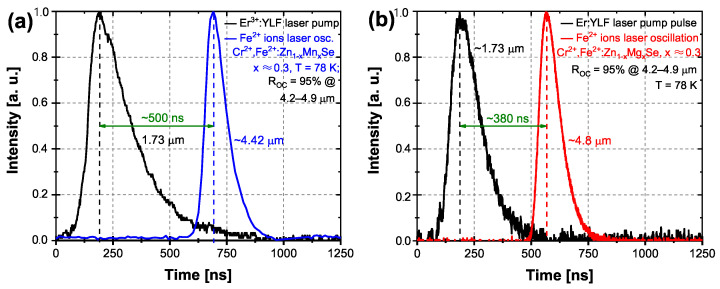
Oscillograms of the Q-switched Er:YLF laser pump pulse at 1.73 μm (black curves) and Fe${}^{2+}$ laser oscillation in the case of the Cr${}^{2+}$→ Fe${}^{2+}$ ions’ energy transfer for the temperature of 78 K. (**a**) Cr,Fe:Zn${}_{1-x}$Mn${}_{x}$Se (x ≈ 0.3)—blue curve. (**b**) Cr,Fe:Zn${}_{1-x}$Mg${}_{x}$Se (x ≈ 0.3)—red curve.

Typical oscillograms of the pump and generated laser pulses for the case of the Cr${}^{2+}$ → Fe${}^{2+}$ ions’ energy transfer are presented in Figure 9. There was a time delay between the Er:YLF laser pumping pulse and the generated Fe${}^{2+}$ laser oscillations pulse depicted by a green arrow. The temporal profile of the Fe${}^{2+}$ ions oscillation of the Mn laser is shown in Figure 9a with a delay of about ∼500 ns and a pulse duration of ∼150 ns. The temporal pulse profile of the Mg laser system is shown in Figure 9b, and the delay between pulses was ∼380 ns before the ∼100 ns Fe${}^{2+}$ ions generated the laser pulse. It should be noted that the Cr${}^{2+}$ ions’ laser oscillations were not observed for both sets of cavity mirrors in these conditions.

**Figure 10:**
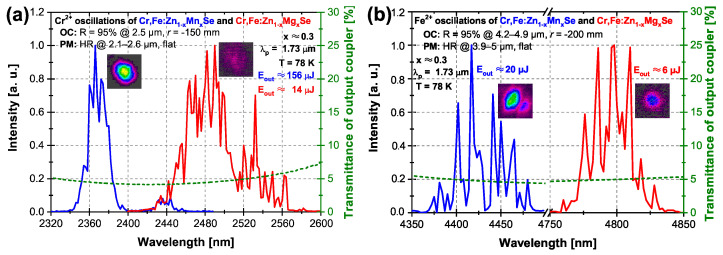
Laser oscillation spectra of the Cr${}^{2+}$ and Fe${}^{2+}$ ions of Cr,Fe:Zn${}_{1-x}$Mn${}_{x}$Se (x ≈ 0.3) (blue curves) and Cr,Fe:Zn${}_{1-x}$Mg${}_{x}$Se (x ≈ 0.3) (red curves) laser systems for the temperature of 78 K, excited at the wavelength of $\lambda_{p}$≈ 1.73 μm presented together with the output coupler transmittance (green curves) and the laser beam profile structure insets. (**a**) Directly excited Cr${}^{2+}$ laser oscillation spectra. (**b**) Fe${}^{2+}$ laser oscillation spectra excited via the Cr${}^{2+}$→ Fe${}^{2+}$ energy transfer.

Figure 10 presents the laser oscillation spectra of Cr${}^{2+}$ (Figure 10a) and Fe${}^{2+}$ (Figure 10b) ions excited by the Er:YLF laser at 1.73 μm with adequate laser cavity mirrors set in each case. In Figure 10a, the oscillation spectra of Cr${}^{2+}$ ions are shown. Oscillations of the Fe${}^{2+}$ ions were not supported by cavity mirrors and thus were not observed. The central laser oscillation wavelength of Cr${}^{2+}$ ions was red-shifted by about 125 nm in the case of the Mg-based crystal (∼2.49 μm) in comparison with the Mn sample (∼2.37 μm). The maximum output energies of ∼156 and ∼14 μJ for Mn and Mg lasers were obtained, respectively.

The Fe${}^{2+}$ ions’ laser oscillation spectra obtained through the Cr${}^{2+}$ → Fe${}^{2+}$ ions’ energy transfer corresponding to temporal profiles in Figure 9 is shown in Figure 10b. The oscillation spectrum maximum of the Mg sample was observed at ∼4.8 μm, which was shifted about ∼380 nm farther toward the mid-infrared part of the optical spectrum with respect to the Mn-containing crystal (∼4.42 μm), similarly to the case of Cr${}^{2+}$ ion oscillations. The laser oscillations obtained are in good agreement with the expectations from the obtained results of the fluorescence spectra measurement (see Figure 3). It is worth noting here that Cr${}^{2+}$ ion oscillations were not observed in this case.

#### 3.2.2. Er:YAG and Fe:ZnSe Laser Excitation at 2.94 and ∼4.05 μm

**Figure 11:**
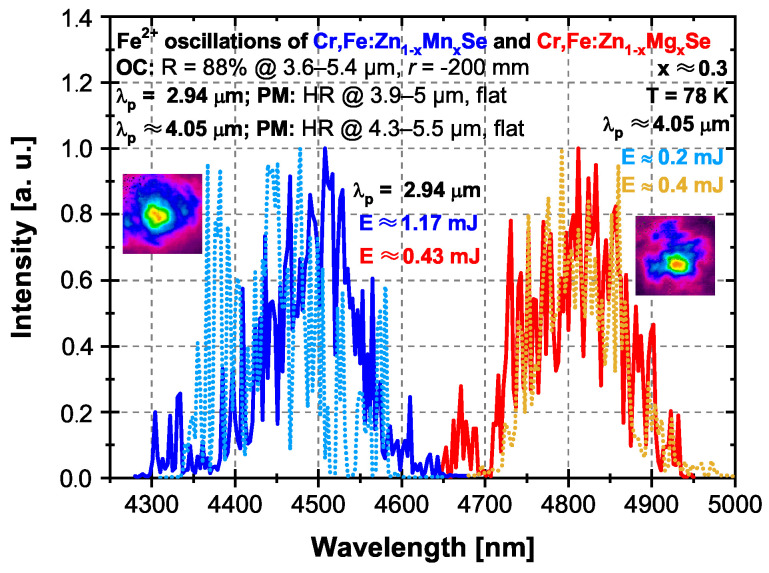
The comparison of the Fe${}^{2+}$ ions’ laser oscillations of the Cr,Fe:Zn${}_{1-x}$Mn${}_{x}$Se and Cr,Fe:Zn${}_{1-x}$Mg${}_{x}$Se (x ≈ 0.3) laser systems for two different direct excitation wavelengths $\lambda_{p}$ = 2.94 μm and $\lambda_{p}$≈ 4.05 μm (all at 78 K). $\lambda_{p}$ = 2.94 μm: Cr,Fe:Zn${}_{1-x}$Mn${}_{x}$Se and Cr,Fe:Zn${}_{1-x}$Mg${}_{x}$Se (x ≈ 0.3)—full blue line and full red line curves, respectively. $\lambda_{p}$≈ 4.05 μm: Cr,Fe:Zn${}_{1-x}$Mn${}_{x}$Se and Cr,Fe:Zn${}_{1-x}$Mg${}_{x}$Se (x ≈ 0.3)—light blue and orange dotted curves, respectively. Inset—the output laser beam profile structures for excitation by 2.94 μm radiation.

For the direct excitation of Fe${}^{2+}$ ions in both crystals, the Q-switched Er:YAG laser was used first. The laser cavity was the same for both samples and consisted of a flat PM: HR at 3.9–5 μm and a curved (*r* = −200 mm) OC with a reflectivity of R${}_{\mathrm{OC}}$ = 88% at 3.6–5.4 μm. The comparison of Fe${}^{2+}$ ions’ laser oscillation spectra under 2.94 and ∼4.05 μm pumping for both samples at 78 K is shown in Figure 11. The laser oscillation spectra were centered around similar wavelengths for both excitation wavelengths used, as can be seen in Figure 11. The maximum intensity of the laser oscillation spectrum was observed at wavelengths of ∼4.51and ∼4.81 μm for Mn and Mg lasers, respectively. These wavelengths were comparable to that observed for the excitation via the Cr${}^{2+}$ → Fe${}^{2+}$ ions’ energy transfer (see Figure 10b). Some differences in the excitation via this mechanism could probably be caused by higher cavity losses (output coupler reflectivity of R${}_{\mathrm{OC}}$ = 88% vs. R${}_{\mathrm{OC}}$ = 95% used for the pumping at 1.73 μm) and lower pumping level. The Mn and Mg lasers’ output energies were ∼1.17 and ∼0.43 mJ for the 2.94 μm radiation excitation, which correspond to optical-to-optical efficiencies of ∼16% and ∼8%, respectively.

Moreover, the gain-switched Fe:ZnSe laser operated at 78 K, generating radiation at ∼4.05 μm, was used for the Fe${}^{2+}$ ions’ direct excitation. The laser cavity was composed of a flat PM: HR at 4.3–5.5 μm and the same OC as in the case of 2.94 μm excitation with a reflectivity of R${}_{\mathrm{OC}}$ = 88% at 3.6–5.4 μm. The Fe${}^{2+}$ ions’ oscillation spectra of both samples under ∼4.05 μm excitation radiation are shown by dashed lines in Figure 11. In the case of Mn and Mg samples pumped at ∼4.05 μm, the output energies of ∼0.2 and ∼0.4 mJ were obtained, corresponding to optical-to-optical efficiencies of ∼24% and ∼8%, respectively. The lower output energy Mn laser system in comparison with 2.94 μm radiation excitation could be explained by the about four-times lower absorption of this crystal at the wavelength of ∼4.05 μm. The central oscillation wavelengths for both samples were similar, as in the case of 1.73 or 2.94 μm excitation radiation used. It is good to note here that any significant shift in fluorescence spectra between 1.73 and 2.94 μm or ∼4.05 μm excitation radiation used was not also observed.

## 4. Discussion

In this paper, we present the comparison of two novel Cr,Fe:Zn${}_{1-x}$Mn${}_{x}$Se and Cr,Fe:Zn${}_{1-x}$Mg${}_{x}$Se (x ≈ 0.3) single crystal-based solid-state laser systems generating laser radiation from Cr${}^{2+}$ and Fe${}^{2+}$ ions within the middle-infrared part of the spectrum around ∼2.5 and ∼4.8 μm. The crystals were gradually pumped by coherent radiation of three different lasers. The Q-switched Er:YLF laser generating radiation at 1.73 μm within the Cr${}^{2+}$ absorption band was used for the direct excitation of Cr${}^{2+}$ ions as well as for the indirect Fe${}^{2+}$ ions excitation via the Cr${}^{2+}$ → Fe${}^{2+}$ ions’ energy transfer. The direct excitation of Fe${}^{2+}$ ions was provided by the Q-switched Er:YAG laser at the wavelength of 2.94 μm or by the gain-switched, LN${}_{2}$-cooled operated Fe:ZnSe laser at ∼4.05 μm. Spectroscopic and laser output properties at 78 K for all types of optical pumping were compared.

The maxima of absorption spectra, as well as whole absorption bands of both active ions, were shifted farther to the mid-infrared part of the optical spectrum in the case of the Mg sample. The difference between the Fe${}^{2+}$ ions’ fluorescence spectra excited via the inter-ionic energy transfer at the wavelength of 1.73 μm or directly at 2.94 μm was almost negligible for both crystals. This may be one of the reasons why both laser systems’ output oscillations were detected around similar central wavelengths for both types of Fe${}^{2+}$ ions’ excitation. The maximal fluorescence signals of the Fe${}^{2+}$ ions fluorescence in Mn and Mg samples were detected at ∼4.05 and ∼4.52 μm at 78 K, respectively.

The temperature has a significant impact on all spectroscopic as well as laser properties of the studied materials (except for Cr${}^{2+}$ ions’ lifetime up to ∼270 K). With a rising temperature from 78 up to 300 K, the whole absorption of both materials decreased by about ∼10% and ∼7% for Mn and Mg samples, respectively. Except for strong Fe${}^{2+}$ lifetime quenching with temperature, this could be one of the reasons why the laser output properties get worse with a rising temperature. A similar change in the fluorescence spectra was observed. Due to this fact, the laser gain of the investigated materials is being shifted farther to the mid-infrared part of the optical spectrum with an increasing temperature, and thus the central laser oscillation wavelength as well.

The fluorescence lifetime of Cr${}^{2+}$ ions (at the decay curve’s tail) was also studied, and the values are ∼5.3 and ∼5.0 μs for Mn and Mg samples at 78 K, respectively. The Cr${}^{2+}$ lifetime slowly decreases with the increasing temperature down to ∼4.2 and ∼4.0 μs at 300 K for Mn and Mg samples, respectively.

The direct excitation of Cr${}^{2+}$ ions by the Er:YLF laser at 1.73 μm with adequate laser cavity mirrors used led to the Cr${}^{2+}$ central oscillations at ∼2.37 and ∼2.49 μm at 78 K with a maximum obtained output energy of ∼156 and ∼14 μJ for lasers based on Mn and Mg co-doped solid solutions, respectively. The corresponding optical-to-optical efficiencies were ∼4% and ∼0.2%, respectively. After the exchange of the laser cavity mirrors, but still for the same pumping conditions, the Cr${}^{2+}$ → Fe${}^{2+}$ ions’ energy transfer was demonstrated. Using this excitation process, the laser output energies were ∼20 μJ at ∼4.42 μm and ∼6 μJ at ∼4.8 μm, both at 78 K for Mn and Mg samples, respectively. The central oscillation wavelength of the Fe${}^{2+}$ and Cr${}^{2+}$ ions was red-shifted for magnesium-containing samples compared to the host material with manganese. The lower output energy from the Mg laser system can be a consequence of lower active ions concentration (of about five times in comparison with the Mn sample), as well as its worse optical quality.

The direct pumping of Fe${}^{2+}$ ions by the Er:YAG laser at the wavelength of 2.94 μm led to Fe${}^{2+}$ ions’ laser oscillations at similar wavelengths as in the previous case of the energy transfer excitation for both samples at 78 K. The Mn and Mg lasers’ output energies were ∼1.17 and ∼0.43 mJ for the 2.94 μm radiation excitation, respectively. The obtained energies correspond to optical-to-optical efficiencies of ∼16% and ∼8%, respectively. The possibility of ∼4.05 μm gain-switched Fe:ZnSe laser excitation of both samples was demonstrated with output energies of ∼0.2 and ∼0.4 mJ, corresponding to optical-to-optical efficiencies of ∼24% and ∼8% for Mn and Mg samples, respectively. The central oscillation wavelengths were localized around ∼4.45 and ∼4.8 μm, similar to the case of the 2.94 μm excitation radiation used.

## 5. Conclusions

In conclusion, both active laser crystalline media demonstrated an opportunity of mid-IR radiation generation under direct Cr${}^{2+}$ ions’ excitation at 1.73 μm to get ∼2.37 and ∼2.49 μm radiation or Fe${}^{2+}$ ions excitation via the Cr${}^{2+}$ → Fe${}^{2+}$ ions’ energy transfer at the same pump wavelength to obtain ∼4.4 or ∼4.8 μm lasing. This principle could reduce the final scale of laser systems generating in a range of 4–5 μm when pumped by laser diodes or fiber lasers and make such mid-IR laser sources more compact in the future. However, in this mode of operation, these materials can still only be used at low temperatures because, with the increasing temperature, the Fe${}^{2+}$ laser output parameters decrease. A reason for this, is most probably caused by the shortening of the Fe${}^{2+}$ ions’ lifetime because of the thermally activated non-radiative quenching. As was shown previously [36,38], the maximal Cr${}^{2+}$ → Fe${}^{2+}$ energy transfer rate was estimated to be ∼10${}^{6}$ s${}^{-1}$, which becomes quite long compared to the Fe${}^{2+}$ ions’ lifetime with a temperature increase (Fe${}^{2+}$ lifetime becomes hundreds of nanoseconds at RT) thus this effect is particularly pronounced in the case of excitation via the Cr${}^{2+}$ → Fe${}^{2+}$ ions’ energy transfer.

Direct pumping of the Fe${}^{2+}$ ions’ absorption band at the wavelengths of 2.94 and ∼4.05 μm was also successfully demonstrated and resulted in even higher output energy due to lower quantum defects and a higher pumping rate. The laser operation in the gain switch mode (a short pulse excitation) is not strongly limited by the temperature of the active laser medium from 78 K up to RT in this case, except for the output energy decrease. Thus, nanosecond pulses with a duration comparable to the Fe${}^{2+}$ ions’ lifetime, which has a tendency for fast temperature-induced shortening, should be used for the pumping. Therefore, an optimization of manganese and magnesium content (x), as well as the relative and absolute concentrations of Cr${}^{2+}$ and Fe${}^{2+}$ active ions in the Cr,Fe:Zn${}_{1-x}$Mn${}_{x}$Se and Cr,Fe:Zn${}_{1-x}$Mg${}_{x}$Se solid solutions, is required to optimize pumping via the Cr${}^{2+}$ → Fe${}^{2+}$ energy transfer process. Moreover, antireflection coatings of crystal faces, better optical quality, optimal output coupling conditions, or cavity mirror placement inside the cryostat chamber may further improve these laser systems’ output characteristics.

## Figures and Tables

**Table 1:** Overview of three laser systems’ properties used for the excitation of crystal samples. RT—room temperature.

Laser; Operation Temperature	Central Osc. Wavelength (μm)	Pulse Duration (FWHM) (ns)	Pulse Energy (mJ)	Repetition Rate (Hz)
(a) Q-switched Er:YLF; RT	1.73	175	∼13	1
(b) Q-switched Er:YAG; RT	2.94	160	∼14	2.5
(c) Fe:ZnSe; 78 K	∼4.05	150	∼9	1

**Table 2:** Absorption spectra properties of Cr,Fe:Zn${}_{1-x}$Mn${}_{x}$Se (x ≈ 0.3) and Cr,Fe:Zn${}_{1-x}$Mg${}_{x}$Se (x ≈ 0.3) crystals at 78 and 300 K.

Crystal Sample	Cr${}^{2+}$ Ions’ Absorption Band FWHM at 78/300 K	Cr${}^{2+}$ Ions Max. Absorption Wavelength at 78/300 K	Fe${}^{2+}$ Ions’ Absorption Band FWHM at 78/300 K	Fe${}^{2+}$ Ions Max. Absorption Wavelength at 78/300 K
**Cr,Fe:Zn${}_{1-x}$Mn${}_{x}$Se (x ≈ 0.3)**	294/386 nm	1774/1782 nm	1060/1460 nm	2930/2950–3200 nm
**Cr,Fe:Zn${}_{1-x}$Mg${}_{x}$Se (x ≈ 0.3)**	370/416 nm	1850/1870 nm	1290/1540 nm	3425/3425 nm

**Table 3:** Absorption coefficient values of Cr,Fe:Zn${}_{1-x}$Mn${}_{x}$Se (x ≈ 0.3) and Cr,Fe:Zn${}_{1-x}$Mg${}_{x}$Se (x ≈ 0.3) crystals at three excitation wavelengths used for temperatures of 78 and 300 K.

Crystal Sample	Thickness (mm)	Absorption Coefficient at 1.73 μm; 78/300 K	Absorption Coefficient at 2.94 μm; 78/300 K	Absorption Coefficient at ∼4.05 μm; 78/300 K
**Cr,Fe:Zn${}_{1-x}$Mn${}_{x}$Se (x ≈ 0.3)**	2.6 mm	4.7/3.3 cm${}^{-1}$	9.0/5.5 cm${}^{-1}$	0.7/2.9 cm${}^{-1}$
**Cr,Fe:Zn${}_{1-x}$Mg${}_{x}$Se (x ≈ 0.3)**	5.0 mm	1.2/1.0 cm${}^{-1}$	2.5/2.5 cm${}^{-1}$	2.8/2.2 cm${}^{-1}$

**Table 4:** Temperature dependence of the Cr${}^{2+}$ decay time in Cr${}^{2+}$,Fe${}^{2+}$ co-doped ($\tau_{\mathrm{Cr},\mathrm{Fe}}$, at 1/e level), and Cr${}^{2+}$ ions’ lifetime ($\tau_{\mathrm{Cr}}$, measured at the tail) in Cr${}^{2+}$-only doped Zn${}_{1-x}$Mn${}_{x}$Se and Zn${}_{1-x}$Mg${}_{x}$Se (x ≈ 0.3) crystal samples under 1.73 μm Q-switched laser excitation. The comparison of Cr${}^{2+}$→ Fe${}^{2+}$ energy transfer efficiencies calculated according to Equations (1) or (2).

Temperature (K)	Crystal Sample, x ≈ 0.3	Fluorescence Decay Time (1/e Level) $\tau_{\mathrm{Cr},\mathrm{Fe}}$ (μs)	Cr${}^{2+}$ Lifetime (Measured at the Tail) $\tau_{\mathrm{Cr},\mathrm{Fe}}$ (μs)	Energy Transfer Efficiency According to Equation (1) (%)	Energy Transfer Efficiency According to Equation (2) (%)
78 K	Cr,Fe(Cr):Zn${}_{1-x}$Mn${}_{x}$Se	1.6	5.3	70	57
	Cr,Fe(Cr):Zn${}_{1-x}$Mg${}_{x}$Se	1.7	5.0	66	53
200 K	Cr,Fe(Cr):Zn${}_{1-x}$Mn${}_{x}$Se	1.3	5.3	75	62
	Cr,Fe(Cr):Zn${}_{1-x}$Mg${}_{x}$Se	1.5	5.0	70	57
300 K	Cr,Fe(Cr):Zn${}_{1-x}$Mn${}_{x}$Se	1.0	4.2	76	67
	Cr,Fe(Cr):Zn${}_{1-x}$Mg${}_{x}$Se	1.0	4.0	75	65

## Data Availability

The data contained within the article are available on request from the corresponding author. No dataset is publicly available online because the research is still ongoing.
